# Performance Analysis of Wearable Dual-Band Patch Antenna Based on EBG and SRR Surfaces

**DOI:** 10.3390/s22145208

**Published:** 2022-07-12

**Authors:** Abdul Wajid, Ashfaq Ahmad, Sadiq Ullah, Dong-you Choi, Faiz Ul Islam

**Affiliations:** 1 Department of Telecommunication Engineering, University of Engineering and Technology Mardan, Mardan 23200, Pakistan; wajiduetpeshawar@gmail.com (A.W.); sadiqullah@uetmardan.edu.pk (S.U.); 2 Communication and Wave Propagation Laboratory, Department of Information and Communication Engineering, Chosun University, Gwangju 61452, Korea; ashfaquetb11@gmail.com; 3 School of Automation, Nanjing University of Science and Technology, Nanjing 210094, China; uet_faiz@yahoo.com

**Keywords:** patch antenna, split-ring resonator (SRR), specific absorption rate (SAR), electromagnetic bandgap (EBG)

## Abstract

This paper presents the performance comparison of a dual-band conventional antenna with a split-ring resonator (SRR)- and electromagnetic bandgap (EBG)-based dual-band design operating at 2.4 GHz and 5.4 GHz. The compactness and dual-frequency operation in the legacy Wi-Fi range of this design make it highly favorable for wearable sensor network-based Internet of Things (IoT) applications. Considering the current need for wearable antennas, wash cotton (with a relative permittivity of 1.51) is used as a substrate material for both conventional and metamaterial-based antennas. The radiation characteristics of the conventional antenna are compared with the EBG and SRR ground planes-based antennas in terms of return loss, gain, and efficiency. It is found that the SRR-based antenna is more efficient in terms of gain and surface wave suppression as well as more compact in comparison with its two counterparts. The compared results are found to be based on two distinct frequency ranges, namely, 2.4 GHz and 5.4 GHz. The suggested SRR-based antenna exhibits improved performance at 5.4 GHz, with gains of 7.39 dbi, bandwidths of 374 MHz, total efficiencies of 64.7%, and HPBWs of 43.2 degrees. The measurements made in bent condition are 6.22 db, 313 MHz, 52.45%, and 22.3 degrees, respectively. The three considered antennas (conventional, EBG-based, and SRR-based) are designed with a compact size to be well-suited for biomedical sensors, and specific absorption rate (SAR) analysis is performed to ensure user safety. In addition, the performance of the proposed antenna under bending conditions is also considered to present a realistic approach for a practical antenna design.

## 1. Introduction

In the modern communication era, the wearable antenna is a topic of interest, with the recent development of several advanced technologies, such as the Internet of Things (IoT), for communications both off-body and on-body to fixed or mobile local wireless networks [1]. These systems are directly attached to the human body or fixed on clothes; they are of high interest for detecting the motion of a body during exercise and for medical applications, such as monitoring of blood pressure and heartbeat, and they form a local network of several sensors using the local Wi-Fi frequency range [2]. The major challenge for the design of wearable antennas is achieving compactness and the required flexibility while maintaining a high quality of service for a standard application. The microstrip patch antenna is a favorable choice for wearable devices, as it is flexible, conformable, lightweight, inexpensive, easy to fabricate, and low-profile. However, it has certain limitations, such as low gain, reduced efficiency, narrow bandwidth, spurious radiation, and out-of-phase reflections [3]. Such worn antennas with high back-lobe radiations may cause increased electromagnetic absorptions when placed close to the body [4]. To overcome these limitations, metamaterial surfaces or artificial ground planes can serve as an alternative. These surfaces aid in the suppression of the surface wave and provision of in-phase reflections to further enhance the antenna performance in terms of gain, directivity, efficiency, and bandwidth of the patch antenna [5]. For the use of metamaterials, Veselago’s work revealed that such materials are artificial and not found in nature [6]. Moreover, as electric permittivity and magnetic permeability characterize a specific material, metamaterials have negative values for either one or both [7]. Among such materials, a split-ring resonator (SRR) is a better choice to achieve optimum surface wave suppression, selectivity, and size miniaturization features in many RF microwave devices [8,9,10]. The SRR was first recommended by Pendry [11] and experimentally demonstrated by Smith et al. [12]. To achieve surface wave suppression, the SRR inhabits double-negative properties, including negative values for permittivity and its permeability being within the operating frequency range of the sub-6 GHz band [13,14,15], while EBG only possesses negative permittivity [16]. Thus, the SRR has an advantage over the EBG, because the phase and the energy of the electromagnetic waves in the double-negative media flow in opposite directions, which makes the wave move in a backward direction. For such interesting electromagnetic characteristics of the SRR there are several applications, including antenna design, electromagnetic cloaking, and SAR reduction [17], and it is considered in this paper for a wearable antenna design.

The SRR is a thin wire structure employed in the design of antenna structures to achieve negative values of effective permittivity and permeability [18]. The SRR has gained eminent attention due to its role in achieving high bandwidth, gain, and coupling reduction [19]. In the past, prominent researchers have researched different types of antenna mounted with SRRs and EBG, where the ground plane figures out in the shape of SRR and EBG metamaterials [20]; with CR-SRR-based negative metamaterial for multiple-frequency operation in communication systems [21]; improvements of gain and efficiency using EBG [22]; and mutual coupling reduction by employing an EBG structure [23]. Surface waves propagate in the antenna structure when the surface current on the patch antennas is excited; by introducing the EBG structure, these waves are suppressed in the desired band [24]. Antennas have been designed for millimeter wave applications, but attenuation due to the higher frequency and line of sight losses (LOS) make them less efficient [25]. Numerous antenna structures backed by EBG have been reported to increase gain and bandwidth and to reduce the SAR (specific absorption rate), such as the printed Yagi-Uda Antenna backed by EBG for on-body communication [26], mono source patch antennas for high-directivity applications [27], and textile antennas inspired with EBG for wearable medical applications [28]. In this design, holes were introduced for shunt inductance, which is difficult to fabricate in practical applications [29]. In contrast to the EBG structure, different researchers have carried out research involving SRRs in the UWB spectrum for dual-band-notched responses [30]; developed SRR sensors to detect the permittivity of liquid [31]; and developed SRR metamaterial for pass band and stop band characteristics [32].

This work extends our previous design in [33] and provides a much improved design. Considering the poor performance of recently reported designs [24,25], due to low gain and bandwidth or lack of practicability in the improved designs in [28,30], the objective of this work is to provide a promising solution for wearable applications. The unique work in this paper consists in the analysis of an EBG structure-based antenna and its comparison with SRR for different design parameters. In terms of a significant contribution, the proposed model of a SRR- and EBG-based antenna shows its dominance in the parameters of gain, bandwidth, and surface wave suppression over a conventional patch antenna. The detailed analysis of the optimized design in terms of flexibility and SAR analysis adds to the novel aspect of our work for wearable antenna applications. It is worth mentioning that EBG suppressed the wave only in one band, while SRR suppressed the wave in both bands.

## 2. Materials and Methods

The design process for the proposed antenna is explained in this section. In the first step, a conventional approach was used to form a general design (termed as “conventional antenna” throughout the paper), which was then improved through the characterization of the EBG and SRR for surface wave suppression in the 2.4 GHz and 5.4 GHz bands.

### 2.1. Conventional Patch Antenna

**Figure 1:**
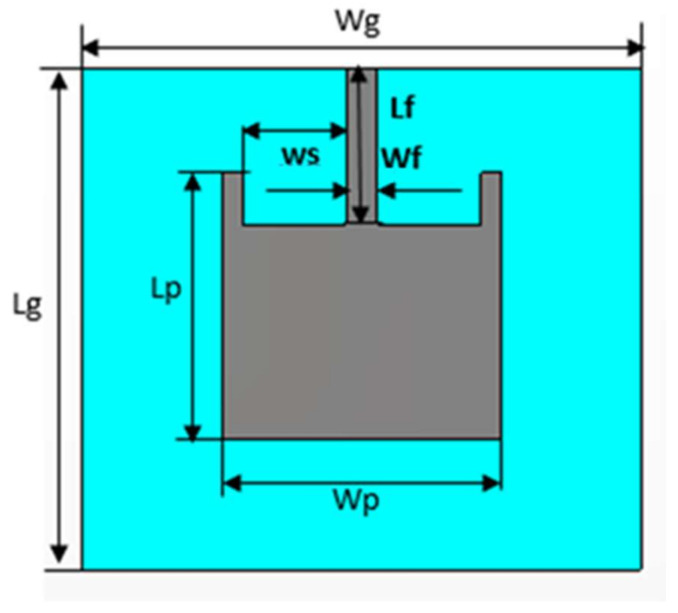
Geometrical representation of the patch antenna (conventional).

The geometry of a conventional microstrip patch dual-frequency operating antenna (for 2.4 GHz and 5.4 GHz) is shown in Figure 1 as designed in CST Microwave Studio (MWS). CST MWS was used to perform simulations for this work because it provides several useful tools for antenna design and analysis. The finite integration-based solver of CST provides a comprehensive analysis of the performance and efficiency of the designed antenna. A wearable material with a relative permittivity value of 1.51 (loss tangent 0.025) was used as a substrate, with 3 mm of thickness. The dimensions of the radiating patch were 52 mm × 54.7 mm × 0.01 mm, representing the length (Lp), width (Wp), and thickness. In terms of volume (total occupation of the antenna), the proposed design was 98 mm × 109.4 mm × 0.01 mm (L × W × mt). The dimensions of the proposed antenna are listed is Table 1. Perfect electric conductor (PEC) conditions were considered for the radiating patch and ground plane of the antenna. Wearable antennas can be made from microstrip antennas. In RF systems, however, loop, PIFA, slot, and dipole-printed are commonly used. Wearable antennas could be used in mobile phones, IoT, seekers, medical systems, 5G communication, HIPER LAN, WLAN, GPS, etc. To the best of our knowledge, this is a compact antenna for an ISM band (2.4 GHz and 5.4 GHz) with a wide 10 dB operational and fractional bandwidth. The proposed antenna shows satisfactory performance in terms of operating bandwidth, gain, and efficiency in the bending scenarios. Additionally, the antenna has promising gain, acceptable bandwidth, and high efficiency in on-body worn scenarios. Moreover, the antenna has a reasonably low SAR value when kept in proximity to the human body.

The main parametric dimensions can be computed using the following equation for the patch antennas [34]:

(1) $$w=\frac{1}{2f_{r}\sqrt{\mu_{0}\varepsilon_{0}}}\sqrt{\frac{2}{\varepsilon_{r}+1}}=\frac{c}{2f_{r}}\sqrt{\frac{2}{\varepsilon_{r}+1}}$$

where *ε_r_* is the relative permittivity of the substrate, *C* is the speed of light, and *f_r_* represents the resonance frequency. The effective dielectric constant ($\varepsilon_{eff}$) can be found as:

(2) $$\;\;\varepsilon_{eff}=\frac{\varepsilon_{r}+1}{2}+\frac{\varepsilon_{r}-1}{2}\left(\frac{1}{\sqrt{1+\frac{12h}{w}}}\right)$$

where *h* represents the thickness of the substrate. Therefore, the actual length of the patch antenna is found by:

(3) $$L=l_{eff}-2\Delta L$$

where Δ*L* represents the extension length and *l_eff_* represent the effective resonance length of the patch.

### 2.2. Design of Metamaterials EBG and SRR

**Figure 2:**
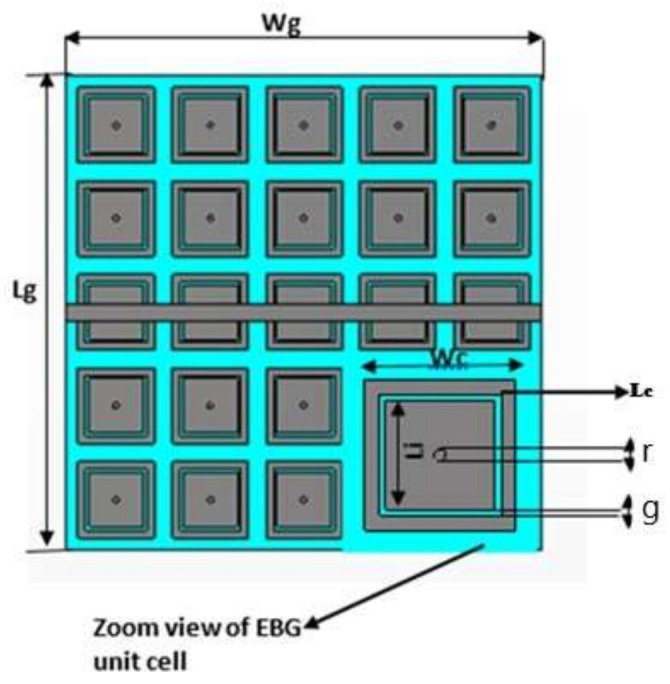
Geometric model of the dual-band EBG.

**Figure 3:**
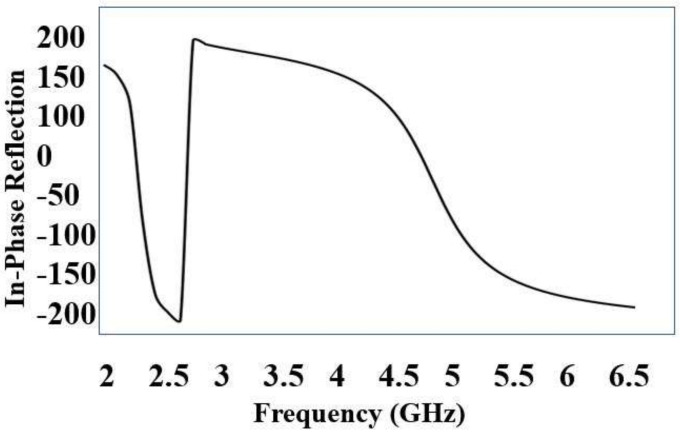
In-phase reflection of EBG unit cell.

A comparison of EBG and SRR structure-based ground planes, as shown in Figure 2 and Figure 3, respectively, is made here by considering 2.4 GHz. Zelt material was used as a conductor, while wash cotton was selected as the dielectric substrate to be compatible with wearable design. The optimized parameters of the proposed EBG- and SRR-based designs, using Sievenpiper’s equations [23], are listed in Table 2 and Table 3, respectively.

#### 2.2.1. Description of EBG Structure

Initially, a dual-band EBG unit cell was designed and analyzed as shown in Figure 2 (zoom view of EBG unit cell); it was observed that the proposed unit cell had in-phase reflections on the desired bands, i.e., 2.4 and 5.4 GHz, as shown in Figure 3. Then, a 5 × 5 array was designed from the proposed unit cells. This surface was characterized in terms of surface wave suppression within a specific resonant bandwidth using the two-port arrangement technique of Figure 2 and fixing a 50 Ω transmission line on the EBG surface to transmit the surface waves. The transmission line is excited at both ends using discrete ports, where one port acts as a source and the other acts as a match load. The transmission coefficient (S21) was, at a minimum, below −35 dB within a desired band at a centered frequency of 2.4 GHz. This clearly depicts that, within this band, the surface wave was suppressed and kept to a minimum level. However, at other desired bands at 5.4 GHz, it lacked the ability to suppress the surface wave. The periodicity of the EBG unit cell is Lp + Gp = 31.4 mm. The via radius was chosen as r = 1 mm. The geometrical model of the dual-band EBG structure is shown in Figure 2.

**Figure 4:**
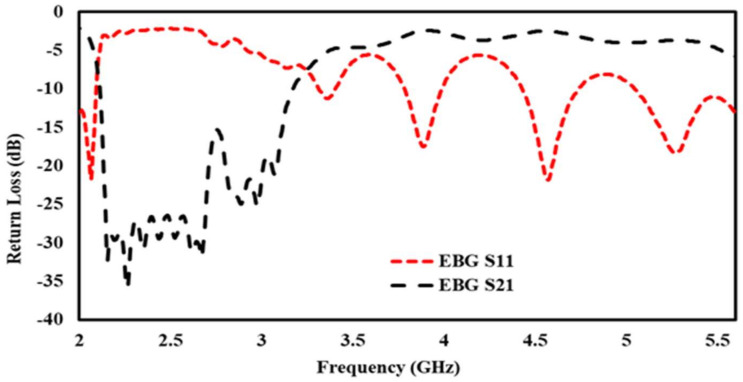
Simulated S11, S21 of EBG array.

This surface was characterized in terms of surface wave suppression within a specific resonant bandwidth using the two-port arrangement technique of Figure 4 and fixing a 50 Ω transmission line on the EBG surface to transmit the surface waves. The transmission line is excited at both ends using discrete ports, where one port acts as a source and the other acts as a match load. The transmission coefficient (S_21_) was, at a minimum, below −35 dB within a desired band at a centered frequency of 2.4 GHz. This clearly depicts that, within this band, the surface wave was suppressed and kept to a minimum level. However, at other desire bands at 5.4 GHz, it lacked the ability to suppress the surface wave. The periodicity of the EBG unit cell is Lp + Gp = 31.4 mm. The via radius was chosen as r = 1 mm. The geometrical model of the dual-band EBG structure is shown in Figure 2.

#### 2.2.2. Description of SRR Structure

**Figure 5:**
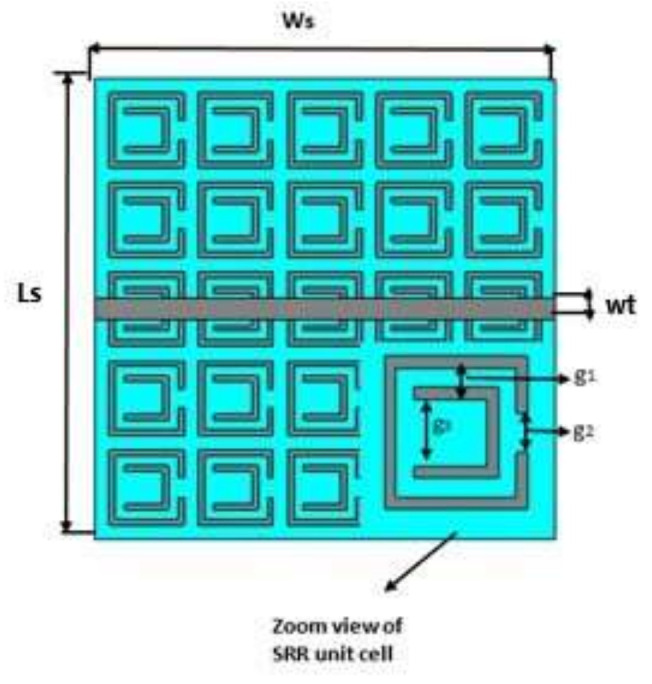
Geometric model of the dual-band SRR.

**Figure 6:**
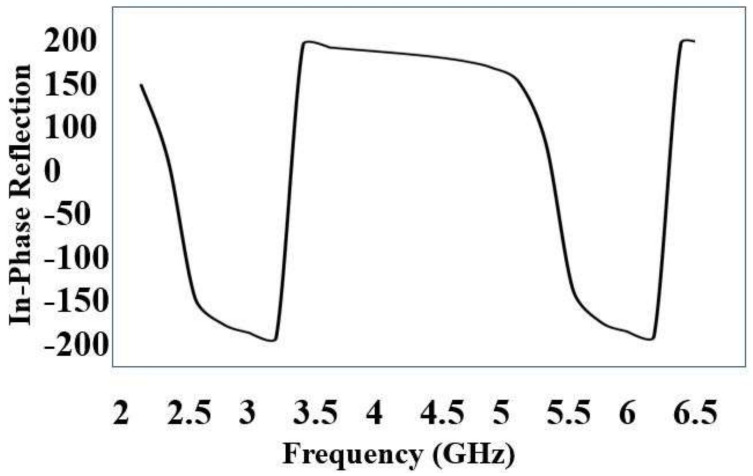
In-phase reflection of SRR unit cell.

Initially, a dual-band SRR unit cell was design and analyzed as shown in Figure 5 (zoom view of SRR unit cell); it was observed that the proposed unit cell had in-phase reflections on the desired bands, i.e., 2.4 and 5.4 GHz, as shown in Figure 6. Then, a 5 × 5 array was designed from the proposed unit cells. The split-ring resonator (SRR) structure proposed in this paper suppresses the surface wave within a specific band of frequencies. The band of frequencies within which the propagation of the wave is highly restricted is called its bandgap. It consists of two rectangular metallic rings and a split on the opposite sides, as shown in Figure 5. Such a design makes it a *LC* resonator (with distributed *L* and *C*).

**Figure 7:**
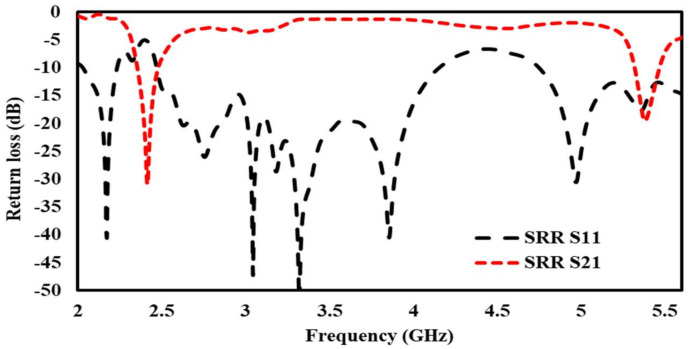
Simulated S11, S21 of 5 × 5 SRR array.

The geometric model of the SRR array is shown in Figure 5. It is worth mentioning that the SRR suppressed the propagation of the wave at both centered frequencies of 2.4 GHz and 5.4 GHz, as shown in Figure 7.

### 2.3. Mushroom-Like EBG and Split-Ring Resonator (SRR)

**Figure 8:**
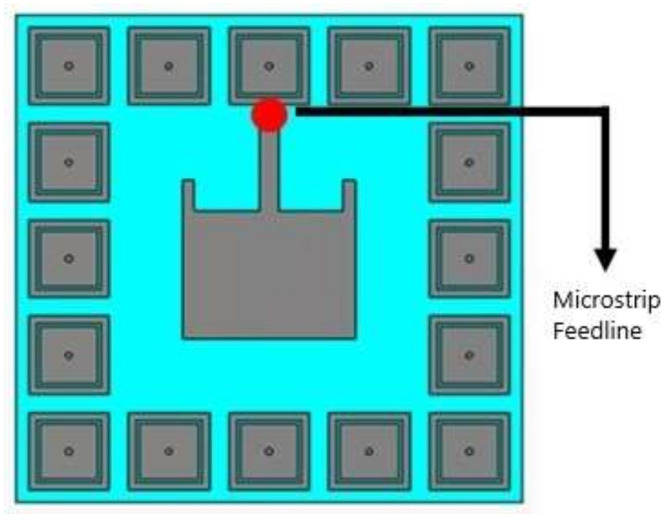
Geometric model of the dual-band EBG-based antenna.

A dual-band microstrip patch antenna was placed above the array of EBG cells by removing a few EBG cells and the transmission line; it was excited by a waveguide port using a microstrip feedline and simulated in CST. The resulting EBG-based antenna shown in Figure 8 operated on dual bands, 2.4 GHz and 5.4 GHz.

**Figure 9:**
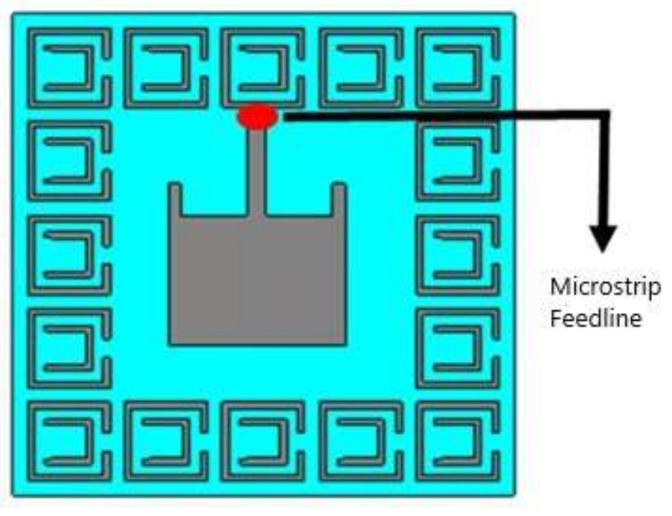
Geometric model (front view) of the dual-band SRR-based antenna.

A dual-band microstrip patch antenna was placed above the array of SRR cells by removing a few SRR cells and the transmission line; it was excited by a waveguide port using a microstrip feedline and simulated in CST. In comparison to wired antennas, wearable antennas are typically low-profile, compact, flexible, lightweight, and inexpensive. The resulting SRR-based antenna shown in Figure 9 operated on dual bands, 2.4 GHz and 5.4 GHz.

### 2.4. Bending Scenario

#### 2.4.1. Bending of Conventional Antenna

**Figure 10:**
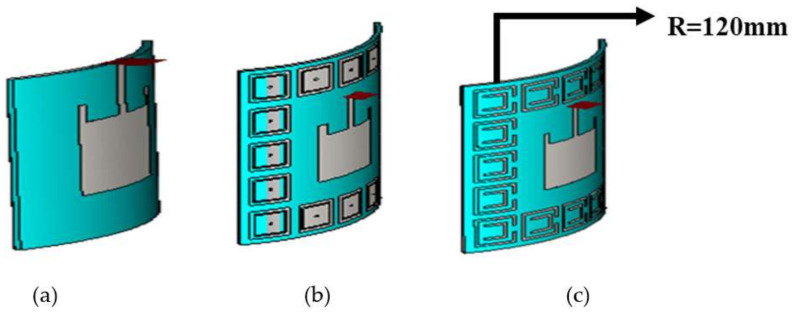
Geometric model of dual-band antennas in free space. (**a**) Conventional antenna. (**b**) EBG-based antenna. (**c**) SRR-based antenna.

For on-body analysis other than flat, a microstrip patch antenna was bent on a 120 mm radius cylinder in CST 2011; we then avoided the cylinder to observe the effect of bending on the previous result and simulated it. The figure of the bent microstrip patch antenna is given below in Figure 10a.

#### 2.4.2. Bending of EBG Antenna

For on-body analysis other than flat, an EBG metamaterial-based microstrip patch antenna was bent on a 120 mm radius cylinder; we then avoided the cylinder to observe the effect of bending on the previous result and simulated it. The geometry of the bent EBG-based patch antenna is shown in Figure 10b.

#### 2.4.3. Bending of SRR Antenna

For on-body analysis other than flat, a SRR metamaterial-based microstrip patch antenna was bent on a 120 mm radius cylinder; we then avoided the cylinder to observe the effect of bending on the previous result and simulated it. The geometry of the bent SRR-based patch antenna is shown in Figure 10c.

#### 2.4.4. Bending of Conventional Antenna on Body

**Figure 11:**
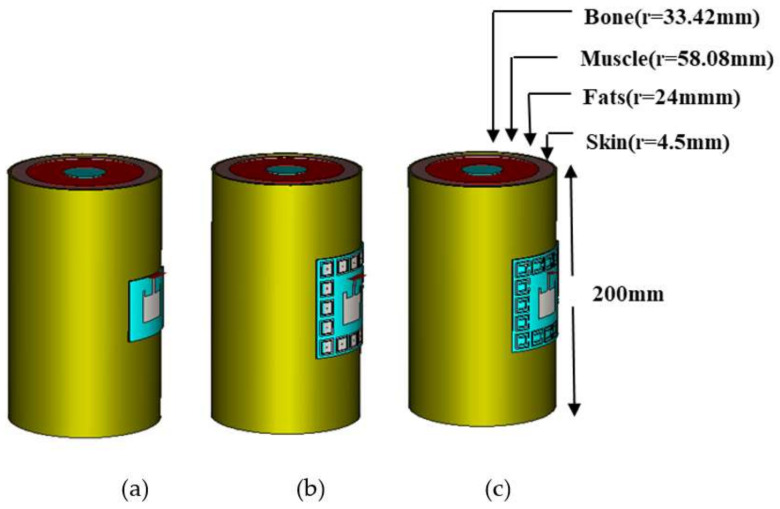
Geometric model of the dual-band antennas bent on human arm. (**a**) Conventional antenna. (**b**) EBG-based antenna. (**c**) SRR-based antenna.

To observe the specific absorption rate (SAR) of a conventional patch antenna, a 120 mm radius arm of the body was designed in CST Microwave Studio which consisted of skin as the upper layer and fat, muscle, and bone as the innermost layer of the body. A conventional patch antenna was placed and bent on the arm of the human body and simulated. The geometry is shown in Figure 11a.

#### 2.4.5. Bending of EBG-Based Antenna on Body

To observe the specific absorption rate (SAR) of an EBG-based patch antenna, a 120 mm radius arm of the body was designed in CST Microwave Studio which consisted of skin as the upper layer and fat, muscle, and bone as the innermost layer of the body. An EBG-based patch antenna was placed and bent on the arm of the human body—the geometry of which is shown in Figure 11b as below—and simulated.

#### 2.4.6. Bending of SRR-Based Antenna on Body

To observe the specific absorption rate (SAR) of a SRR-based patch antenna, a 120 mm radius arm of the body was designed in CST Microwave Studio which consisted of skin as the upper layer and fat, muscle, and bone as the innermost layer of the body. A SRR-based patch antenna was placed and bent on the arm of the human body and simulated. The geometry of the SRR-based patch antenna on the arm is given in Figure 11c. The intrinsic properties of each layer are summarized in Table 4.

## 3. Results and Discussion

The results of the different scenarios (Normal, OFF Body, ON Body) are presented here in terms of their return loss comparison and 2D and 3D antenna radiation pattern.

### 3.1. Normal Scenario

**Figure 12:**
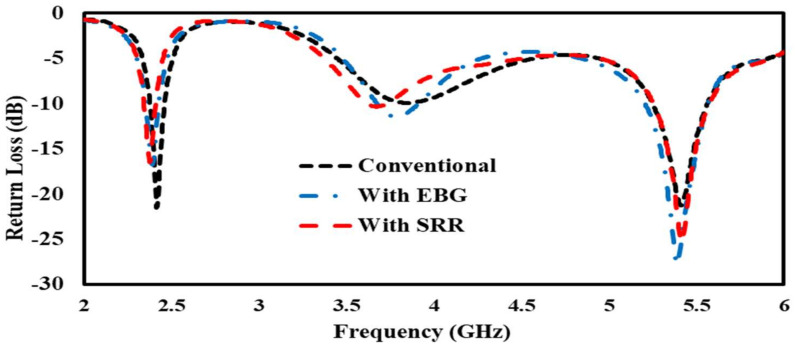
Reflection coefficient (S11) comparison of the conventional antenna with EBG- and SRR--based antennas.

**Figure 13:**
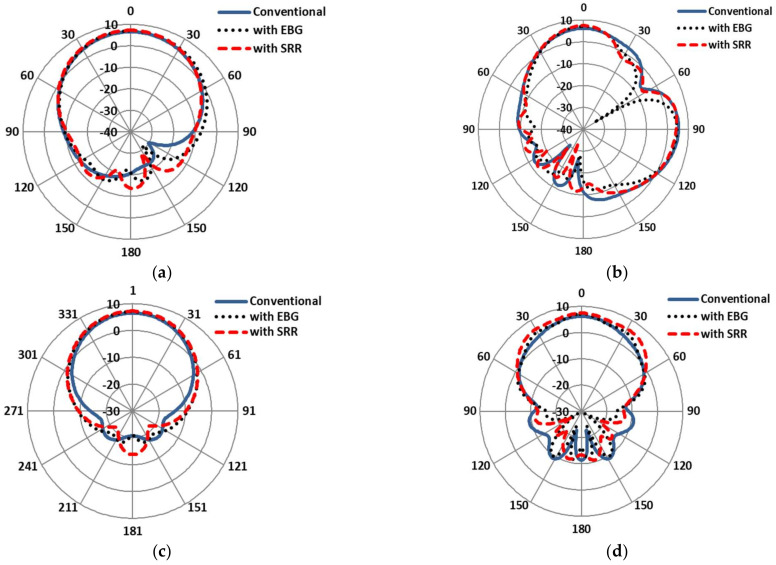
Gain patterns comparisons of conventional antenna with EBG- and SRR-based antennas under normal conditions. (**a**) E-plane at 2.4 GHz. (**b**) E-plane at 5.4 GHz. (**c**) H-plane at 2.4 GHz. (**d**) H-plane at 5.4 GHz.

The results of the normal scenario are discussed in Figure 12 and Figure 13.

#### 3.1.1. Return Loss

Return loss is usually considered as the ratio between reflected and incident power, and it measures how much power is delivered to the antenna. Figure 12 shows a comparison of a conventional patch antenna, EBG-based patch antenna, and SRR-based patch antenna through reflection coefficient plots. The resonance frequencies for all of the types of antenna are 2.4 GHz and 5.4 GHz, shown in Figure 12.

#### 3.1.2. 2D and 3D Gain Plots

The E-plane and H-plane on-body simulated gains of the antennas are compared in Figure 13. It is clearly shown that the main lobe radiation of the SRR-based antenna was maximal, at 7.22 dB, as compared to the EBG-based (7 dB) and conventional patch antennas (6.55 dB) at 2.4 GHz. In addition, the main lobe radiation of the SRR-based antenna was highest, at 6.9 dB, as compared to the EBG-based (6.4 dB) and conventional patch antennas (6.2 dB) at 5.4 GHz. Similarly, the −3dB beamwidth of the SRR-based antenna decayed up to (62.2 deg) relative to the EBG-based (66.00 deg) and conventional patch antennas (71.5 deg) at 2.4 GHz. At the other band of the antenna, the −3 dB beamwidth of the SRR-based antenna was highest (43.2 deg) relative to the EBG-based (36.2 deg) and conventional patch antennas (42.1 deg) at 5.4 GHz.

**Figure 14:**
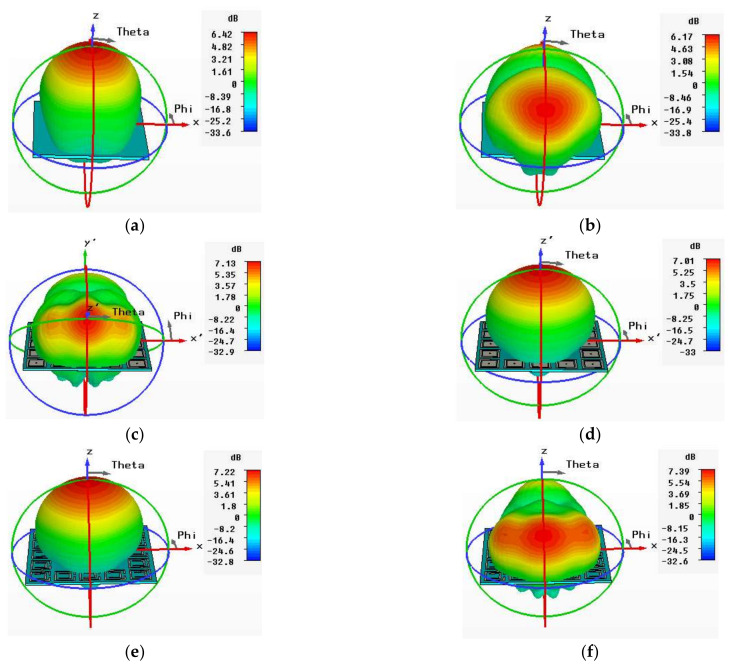
3D radiation pattern of normal scenario. (**a**) Conventional antenna at 2.4 GHz. (**b**) Conventional antenna at 5.4 GHz. (**c**) EBG-based antenna at 2.4 GHz. (**d**) EBG-based antenna at 5.4 GHz. (**e**) SRR-based antenna at 2.4 GHz. (**f**) SRR-based antenna at 5.4 GHz.

The 3D gains of the normal conventional patch and EBG- and SRR-based antennas are shown in Figure 14. The gains of the conventional patch and EBG- SRR-based antennas were 6.55 dB, 7.01 dB, and 7.22 dB, respectively, at 2.4 GHz. Similarly, the gains of the conventional patch and EBG- and SRR-based antennas were 6.17 dB, 7.13 dB, and 7.39 dB, respectively, at 5.4 GHz. The 3D gain patterns of the antennas in the normal scenario are presented in Figure 14a–f. The comparative results show that the SRR-based antenna achieved enhanced results in terms of gains, bandwidth, efficiency, and HPBW at 5.4 GHz. The detailed comparative results are listed in Table 5.

### 3.2. Bent Scenario

#### 3.2.1. OFF Body Analysis

The bent EBG- and SRR-based antennas are compared here and analyzed in the absence of a human arm with a conventional patch antenna using CST Microwave Studio 2011.

#### 3.2.2. Return Losses

**Figure 15:**
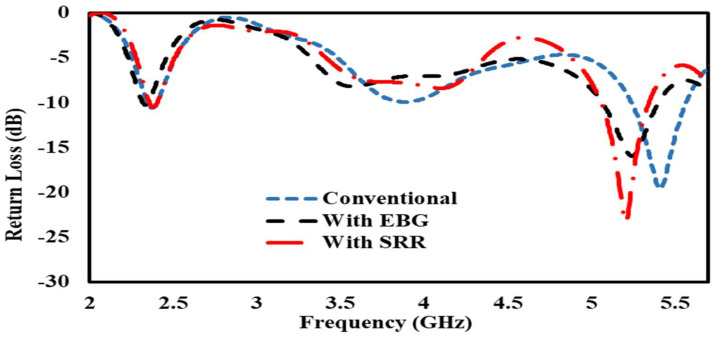
S11 comparison of conventional antenna, antenna on EBG, and SRR/HIS in free space.

Bending affects the impedance of the conventional patch antenna and the EBG- and SRR-based antennas, and the resonance frequencies in all of the antennas slightly shifted toward the left and right from their basic resonance frequencies of 2.4 GHz and 5.4 GHz, as shown in Figure 15.

#### 3.2.3. 2D and 3D Gain Plots

**Figure 16:**
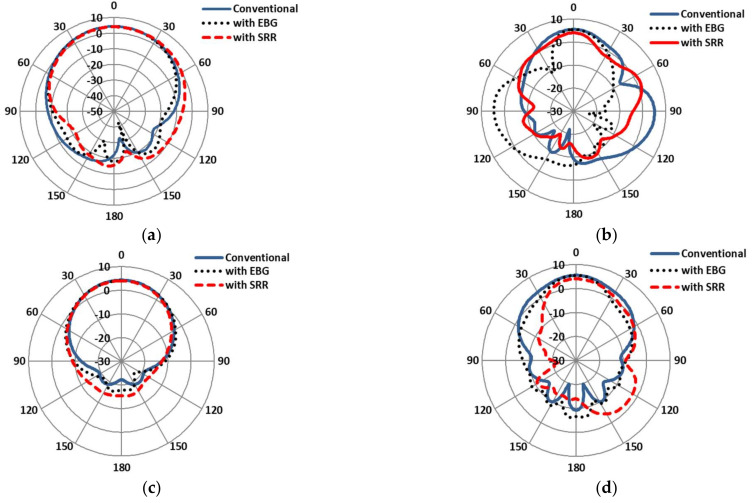
Gain patterns comparisons of conventional antenna under bent condition (free space). (**a**) E-plane at 2.4 GHz. (**b**) E-plane at 5.4 GHz. (**c**) H-plane at 2.4 GHz. (**d**) H-plane at 5.4 GHz.

The off-body E-plane and H-plane simulated gains of the antennas are compared in Figure 16. It can be seen that the main lobe radiation of the SRR-based antenna was maximal, at 4.1 dB, as compared to the EBG-based (3.7 dB) and conventional patch antennas (4.1 dB) at 2.4 GHz. In addition, the main lobe radiation of the SRR-based antenna was highest, at 6.2 dB, as compared to the EBG-based (5.4 dB) and conventional patch antennas (5.5 dB) at 5.4 GHz. Similarly, the −3 dB beamwidth of the SRR-based antenna grew up to (81.2 deg) relative to the EBG-based (73.9 deg) and conventional patch antennas (76.3 deg) at 2.4 GHz. In addition, the −3 dB beamwidth of the conventional patch antenna was highest (47.4 deg) relative to the EBG-based (32.7 deg) and SRR-based antennas (37.5 deg) at 5.4 GHz. The simulated gains comparison of conventional and EBG- and SRR-based antennas at 2.4 GHz and 5.4 GHz is shown in Figure 16a–d.

**Figure 17:**
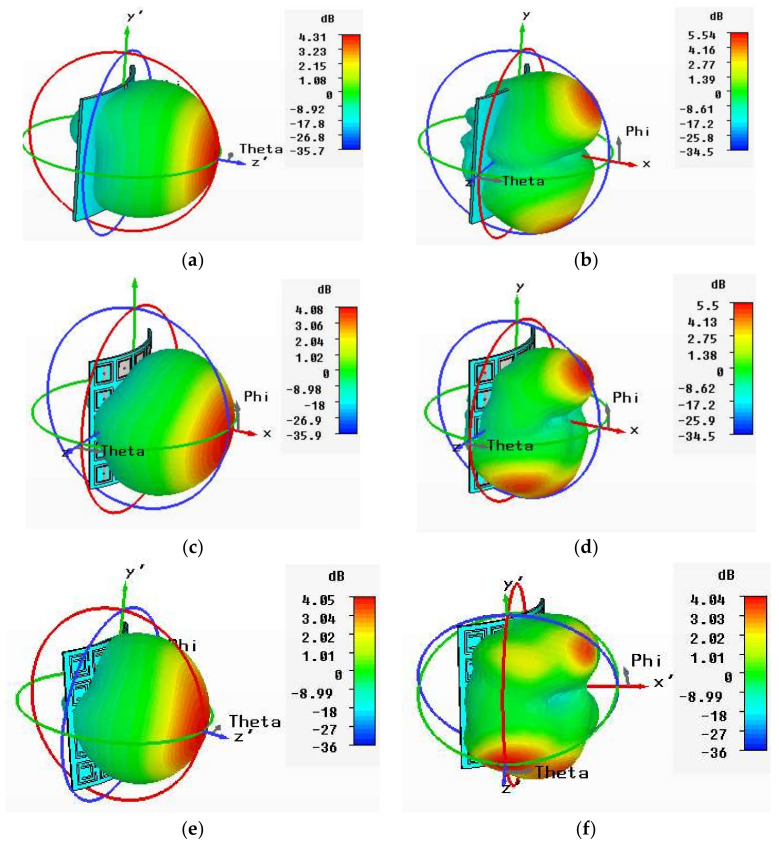
3D radiation pattern of bent scenario (free space). (**a**) Conventional antenna at 2.4 GHz. (**b**) Conventional antenna at 5.4 GHz. (**c**) EBG-based antenna at 2.4 GHz. (**d**) EBG-based antenna at 5.4 GHz. (**e**) SRR-based antenna at 2.4 GHz. (**f**) SRR-based antenna at 5.4 GHz.

The 3D gains of normal conventional patch and EBG- and SRR-based antennas are shown in Figure 17. The gains of the conventional patch and the EBG- and SRR-based antennas are 4.31 dB, 4.08 dB, and 4.05 dB, respectively, at 2.4 GHz. Similarly, the gains of the conventional patch and the EBG- and SRR-based antennas are 5.54 dB, 5.5 dB, and 4.04 dB, respectively, at 5.4 GHz. Table 6 depicts that, comparatively, the boresight gain of the antenna does not show a good result, due to mismatching of the impedance owing to free space. The 3D gain patterns of antennas in free space are presented in Figure 17a–f.

### 3.3. ON Body Analysis

For practical applications, to observe results for conventional and EBG- and SRR-based antennas, a model of the human arm was proposed with a radius of 120 mm. It consisted of four layers: skin, fat, muscle, and bone.

#### 3.3.1. Return Losses

**Figure 18:**
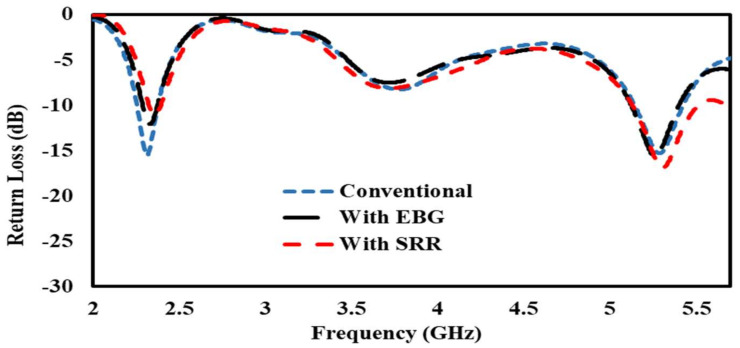
Return loss comparison of conventional antenna, antenna on EBG, and SRR/HIS bent on human arm.

The high-loss nature of the human body lowers the radiating efficiency of a mounted antenna. This section provides the simulation results in terms of return losses and input impedance for a scenario where the proposed EBG- and SRR-based antennas are mounted over a human arm. For comparison, the results from a conventional patch antenna are also presented. The bending of the antenna patch is an important factor to be considered, as it causes a significant reduction in the driving point of the antenna impedance. This results in a small change in the resonant frequency and return loss. Figure 18 shows that the metamaterial-inspired antenna performed very well as compared to the conventional patch antenna in providing low return loss with bending.

#### 3.3.2. 2D and 3D Gain Plots

**Figure 19:**
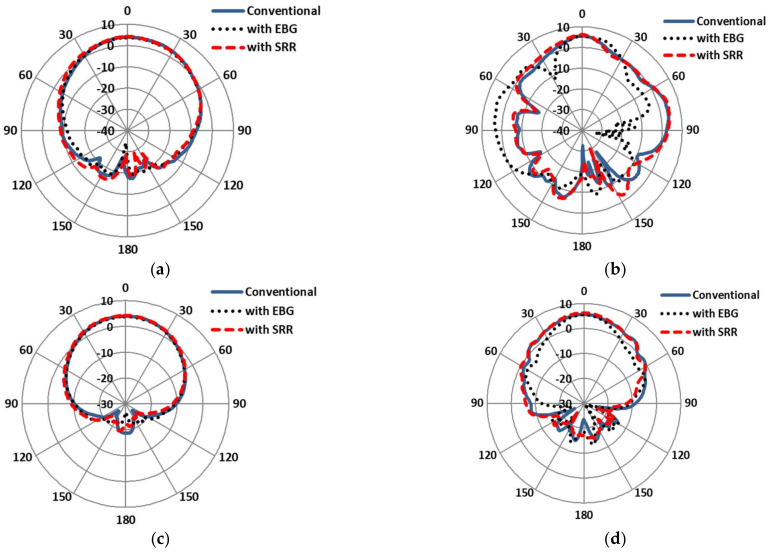
Gain patterns comparisons of conventional antenna under bent condition (on-body). (**a**) E-plane at 2.4 GHz. (**b**) E-plane at 5.4 GHz. (**c**) H-plane at 2.4 GHz. (**d**) H-plane at 5.4 GHz.

The E-plane and H-plane on-body gains of the antennas are compared in Figure 19. The simulation results show that the main lobe radiation of the EBG-based antenna was minimal, at 3.79 dB, as compared to the SRR-based (4.21 dB) and conventional patch antennas (4.03 dB) at 2.4 GHz. In addition, the main lobe radiation of the SRR-based antenna was highest, at 6.22 dB, as compared to the EBG-based (5.39 dB) and conventional patch antennas (5.56 dB) at 5.4 GHz. Similarly, the −3 dB beamwidth of the EBG-based antenna grew up to (89.8 deg) relative to the SRR-based (85.7 deg) and conventional patch antennas (83.0 deg) at 2.4 GHz. In addition, the −3 dB beamwidth of the SRR-based antenna decayed up to (22.3 deg) relative to the EBG-based (39.8 deg) and conventional patch antennas (30.5 deg) at 5.4 GHz. The simulated gains comparison of the conventional and the EBG- and SRR-based antennas at 2.4 GHz and 5.4 GHz is shown in Figure 19a–d.

**Figure 20:**
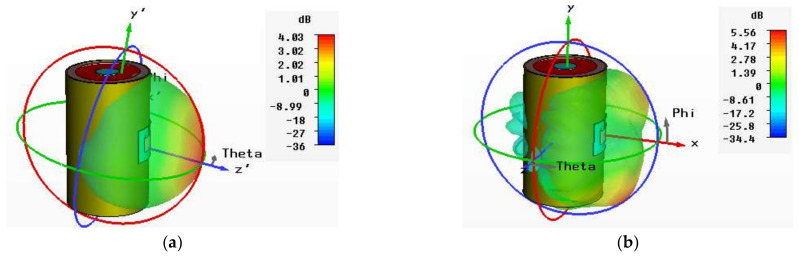

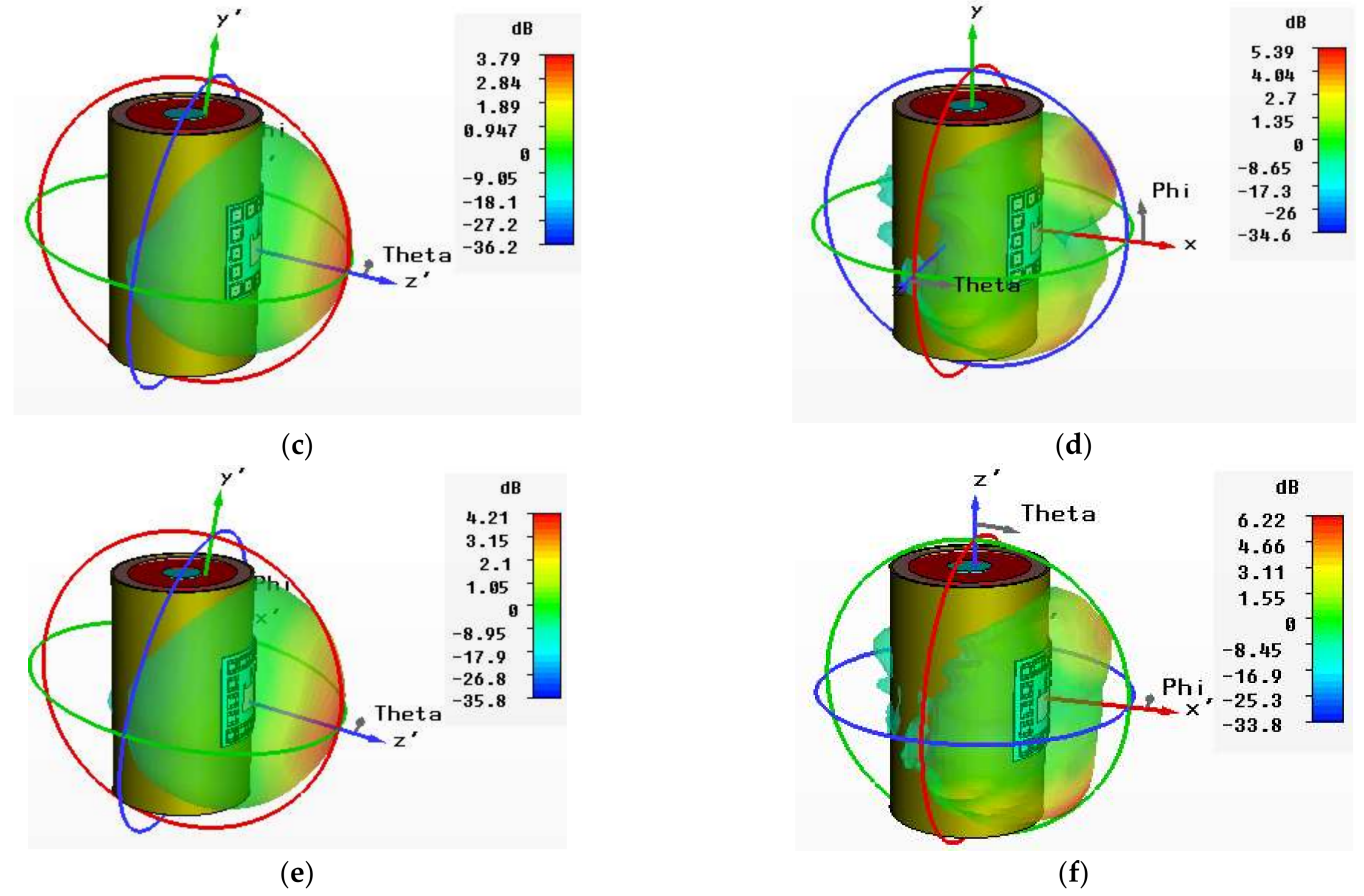
3D radiation patterns in bent scenarios (on-body). (**a**) Conventional antenna at 2.4 GHz. (**b**) Conventional antenna at 5.4 GHz. (**c**) EBG-based antenna at 2.4 GHz. (**d**) EBG-based antenna at 5.4 GHz. (**e**) SRR-based antenna at 2.4 GHz. (**f**) SRR-based antenna at 5.4 GHz.

The 3D gains of the normal conventional patch and the EBG- and SRR-based antennas are shown in Figure 20. The gains of the conventional patch and EBG- and SRR-based antennas are 4.03 dB, 3.79 dB, and 4.21 dB, respectively, at 2.4 GHz. Similarly, the gains of the conventional patch and the EBG- and SRR-based antennas are 5.56 dB, 5.39 dB, and 6.22 dB, respectively, at 5.4 GHz. Table 7 illustrates that the comparative results of the SRR-based antenna is maximal in terms of gain, bandwidth, and total efficiency at 5.4 GHz. The 3D gain patterns of the conventional and EBG- and SRR-based antennas at 2.4 GHz and 5.4 GHz are presented in Figure 20a–f.

## 4. SAR Analysis

For the proposed design of a dual-band antenna, in order to demonstrate its compatibility for human-wearable applications, specific absorption analysis (SAR) was conducted by considering an arm of 200 mm radius. The bending was considered in E-plane in CST MW Studio using the IEEE C95.3 averaging method. The ICNIRP EU standard of 10 g of tissue volume was considered during this analysis. As the human body consists of several conductive and dielectric substances, by bringing an antenna in proximity to the body with an imperfect impedance matching, the human body detunes and reflects backward the radiated energy and then absorbs some part of it. This results in high SAR values, and the use of a high-impedance metamaterial surface as the ground serves as an isolation sheet between the human body and the radiating surface.

### Simulation Setup and SAR Calculation

**Figure 21:**
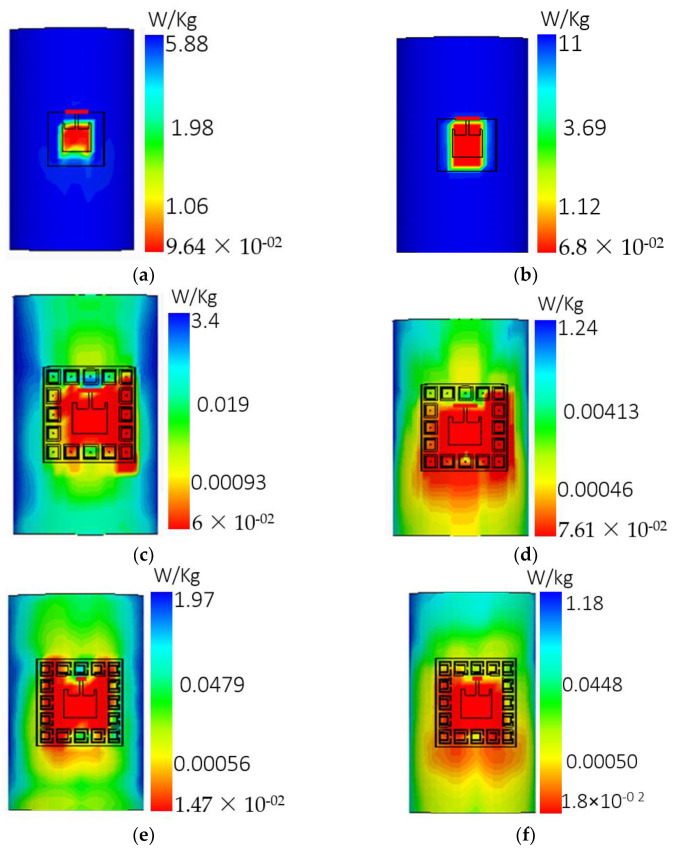
Conventional antenna bent around human arm: (**a**) SAR at 2.4 GHz. (**b**) SAR at 5.4 GHz, EBG-based antenna bent around human arm. (**c**) SAR at 2.4 GHz. (**d**) SAR at 5.4 GHz, SRR-based antenna bent around human arm. (**e**) SAR at 2.4 GHz. (**f**) SAR at 5.4 GHz.

It is clearly shown in Figure 21 that most of the power was reflected and absorbed in the human body in an unloaded metamaterial antenna; therefore, the SAR value at 2.4 GHz was 11 W/kg and 5.88 W/kg at 5.4 GHz, which is above a safe level. Similarly, in the case of the EBG metamaterial-based antenna, the SAR value was 3.40 W/kg at 2.4 GHz and 1.24 W/kg at 5.4 GHz. In the case of the SRR metamaterial-based antenna, the SAR value was 1.97 w/kg at 2.4 GHz and 1.18 w/kg at 5.4 GHz, which is below a safe level (European standard: 2 W/kg). The complete SAR comparison is listed in Table 8. A significant reduction in SAR values was obtained through the metamaterial-inspired design of the antenna because of the HIS (high-impedance surface) when the metamaterial surface was utilized for the ground plane. With such a configuration, resonance at the desired frequency was obtained due to the surface wave bandgap. In addition, this surface also insulates the antenna from electromagnetic radiations from the body.

Various works have been carried out in the past for the enhancement of parameters such as gain, bandwidth, and efficiency, etc. Some of the concluded data from the past works [35,36,37,38] are summarized in the Table 9. It is deduced from the table that the proposed antenna shows supremacy, with reference to the previous work carried out, in terms of gain, bandwidth, and radiation efficiency.

## 5. Conclusions

In this paper, a conventional and metamaterial (EBG, SRR)-based flexible dual-band (2.4 GHz, 5.4 GHz) wearable patch antenna design is presented and analyzed in terms of radiation characteristics. A signigicant contribution is made in this paper with the proposal of a flexible and conformable antenna design for biomedical wearable applications by using a clothing material (wash cotton) as a dielectric. It was observed that the comparative results were based on two different frequency ranges, i.e., 2.4 GHz and 5.4 GHz. At 5.4 GHz, the proposed SRR-based antenna showed enhanced results in terms of gain, bandwidth, total efficiency, and HPBW, which were 7.39 db, 374 MHz, 64.77%, and 43.2 deg, respectively, while, in bent condition, the obtained results were 6.22 db, 313 MHz, 52.45%, and 22.3 deg, respectively. The metamaterial surfaces (EBG and SRR) are proposed to be used as a high-impedance surface for protection from the risks of electromagnetic radiations, and the results show that the SAR values were successfully reduced to the safe limit by employing the metamaterial surfaces. It was observed that, in terms of gain, the SRR-based antenna performance was more dominant compared to the conventional and EBG-based antennas. The performance of the three proposed antennas was evaluated in a bent condition. It was demonstrated that the proposed design offered a great amount of flexibility, and bending had no prominent effects on the performance of the recommended antennas. However, the proposed design has a limited bandwidth, low power-handling capacity, and high power loss at high frequencies, i.e., >12 GHz. Additionally, due to the unavalibility of the fabrication facilities, measured results are not included in the current version.

## Figures and Tables

**Table 1:** Summary of antenna dimensions.

Parameter	Description	Values (mm)
L_P_	Length of patch	52
W_P_	Width of patch	54.7
L_S_	Length of substrate	98
W_S_	Width of substrate	109.4
H	Thickness of substrate	3
mt	Thickness of patch	0.01
W_S_	Width of slot	20.35
S_L_	Depth of slot	10
W_f_	Width of feeder	6
L_f_	Length of feeder	30.25

**Table 2:** Optimized parameter values for the array EBG model.

Parameter	Description	Value (mm)
Wc	Width or length of unit cell	29.4
G	Space b/w adjacent cells	2
R	Via radius	1
Lc	Length of outer ring of EBG unit cell	5.525
A	Periodicity	31.4
Li	Length of inner patch	21.875

**Table 3:** Optimized parameter values for the array SRR model.

Parameters	Descriptions	Value (mm)
g1	Adjacent ring gap	4.5 mm
g3	Inner ring gap	11 mm
g2	Outer split	5.7 mm
A	Periodicity	30.5 mm
L	Width/length of cell	25.5

**Table 4:** Properties of human body tissues.

Tissue	Permittivity (ε_r_)	Conductivity (S/m)	Density (Kg/m^3^)
Skin	35.61	3.2185	1100
Fat	4.6023	0.58521	1100
Muscle	49.278	4.2669	1060
Bone	9.946	1.0101	1850

**Table 5:** Comparative results of (conventional vs. EBG-based vs. SRR-based) antennas in normal scenario.

Parameters	Conventional	With EBG	With SRR	Conventional	With EBG	With SRR
Frequency (GHz)	2.4	2.4	2.4	5.4	5.4	5.4
Gain (dB)	6.55	7.01	7.22	6.17	7.13	7.39
Bandwidth (MHz)	82	77	79	308	360	374
Total Efficiency (%)	60.25	64.52	62.28	62.6	59.2	64.77
HPBW (deg)	71.2	66.00	62.2	42.1	36.2	43.2

**Table 6:** Summary of results (conventional vs. EBG-based vs. SRR-based) bent antennas in free space.

Parameters	Conventional	With EBG	With SRR	Conventional	With EBG	With SRR
Frequency (GHz)	2.37	2.33	2.38	5.41	5.24	5.44
Gain (dB)	4.31	4.08	4.05	5.54	5.5	4.04
Bandwidth (MHz)	55.5	36.27	58	324	333.8	287
Total Efficiency (%)	36.4	26.9	34.5	58.00	50.38	41.5
HPBW (deg)	76.3	73.9	101.8	47.4	32.37	34.5

**Table 7:** Summary of results (conventional vs. EBG-based vs. SRR-based) of bent antennas on human arm.

Parameters	Conventional	With EBG	With SRR	Conventional	With EBG	With SRR
Frequency (GHz)	2.4	2.4	2.4	5.4	5.4	5.4
Gain (dB)	4.03	3.79	4.21	5.56	5.39	6.22
Bandwidth (MHz)	223	62.9	128	286.5	267	313
Total Efficiency (%)	23.34	32.58	37.30	41.2	49.8	52.45
HPBW (deg)	83.00	89.8	85.7	30.5	39.8	22.3

**Table 8:** Summary of simulated SAR normalized to 0.5 W (r.m.s.) at 2.4 GHz and 5.4 GHz.

Antenna	10 g Peak SAR (W/Kg) at 2.4 GHz	10 g Peak SAR (W/Kg) at 5.4 GHz
Conventional	11	5.88
EBG-based	3.40	1.24
SRR-based	1.97	1.18

**Table 9:** Comparison table.

Ref. No	Antenna Size (mm^2^)	Operating Frequency	Gain (db/dbi)	Bandwidth	Efficiency
[35]	100 × 100	2.42	2.42 db	298 MHz	40%
[36]	50 × 50	2.4 ISM	4.12 dbi	65 MHz	78.97%
[37]	102 × 102	2.4/5.5	5.2 db	55 MHz	83%
[38]	84 × 162.25	1.85 -3.15	3.38 db	130 MHz	NA
Proposed work	98 × 109.4	2.4/5.4	7.13/7.39 db	374 MHz	64.77
